# Can frequent long stimulus onset ansynchronies (SOAs) foster the representation of two separated task-sets in dual-tasking?

**DOI:** 10.1007/s00426-024-01935-y

**Published:** 2024-02-28

**Authors:** Lasse Pelzer, Christoph Naefgen, Julius Herzig, Robert Gaschler, Hilde Haider

**Affiliations:** 1 Department of Psychology, University of Cologne, Richard-Strauss-Str. 2, 50931 Cologne, Germany; 2 Department of Psychology, FernUniversität in Hagen, Hagen, Germany

## Abstract

Recent findings suggest that in dual-tasking the elements of the two tasks are associated across tasks and are stored in a conjoint memory episode, meaning that the tasks are not represented as isolated task-sets. In the current study, we tested whether frequent long stimulus onset ansynchronies (SOAs) can foster the representation of two separated task-sets thereby reducing or even hindering participants to generate conjoint memory episodes—compared to an integrated task-set representation induced by frequent short SOAs. Alternatively, it is conceivable that conjoint memory episodes are an inevitable consequence of presenting two tasks within a single trial. In two dual-task experiments, we tested between consecutive trials whether repeating the stimulus–response bindings of both tasks would lead to faster responses than repeating only one of the two tasks’ stimulus–response bindings. The dual-task consisted of a visual-manual search task (VST) and an auditory-manual discrimination task (ADT). Overall, the results suggest that, after processing two tasks within a single trial, generating a conjoint memory episode seems to be a default process, regardless of SOA frequency. However, the respective SOA frequency affected the participants’ strategy to group the processing of the two tasks or not, thereby modulating the impact of the reactivated memory episode on task performance.

## Introduction

In everyday life, it is quite usual to do more than one thing at a time. For instance, people phone while biking or driving a car. Also, things like making breakfast, doing laundry, or shopping often require us to do multiple tasks simultaneously. Thus, multi-tasking is omnipresent in our daily life. Unfortunately, it usually comes with a performance cost (for a current review see Koch et al., 2018).

In the cognitive dual-tasking literature, different sources for these performance costs are discussed. At least three currently dominant classes of models aim to explain these dual-task costs: Bottleneck models, capacity sharing models, and strategic models. From the perspective of bottleneck models, the response selection process is thought to be structurally limited, repressing one task until the response selection process of the other task has finished (Pashler, 1994). In contrast to these models, in capacity sharing models, it is supposed that the response selection can run in parallel, but a limited pool of central resources has to be shared between both tasks which again causes a processing limitation at the response selection stage (Kahneman, 1973; Navon & Miller, 2002; Telford, 1931; Tombu & Jolicoeur, 2003; Welford, 1952). According to the third alternative, strategic models, it is assumed that parallel processing is possible, but serial processing of the two tasks reflects a strategic adaptation to avoid crosstalk between them (Logan & Gordon, 2001; Meyer & Kieras, 1997).

Despite crucial differences, such as the assumption of strictly serial processing versus the possibility of parallel processing, these classes of models mainly focus on processing limitations during the response selection stage as the source of dual-task interference. Recently, the focus on dual-task costs changed a little, by asking how dual-tasks are represented. It is by no means clear whether the participants handle the two tasks as two isolated task-sets or whether they integrate them into one single task-set (Künzell et al., 2018; Schneider & Logan, 2014). In support of such task-integration, it has been shown that the preceding trial shapes the processing of the next trial (De Jong, 1995; Luria & Meiran, 2006). Other observations suggest that even the elements of the two tasks presented in a dual-tasking trial become associated (Schmidtke & Heuer, 1997). It may in fact be natural that the two tasks are not represented separately. The goal of the current study is to contribute further to the understanding of the processes underlying such task integration within dual-tasking trials.

### Task integration in dual tasking

The understanding of the term “Task-Integration” differs among researchers. For example, Ruthruff et al. (2006) and also Schubert et al. (2017) compare dual-tasking costs (the size of the PRP-effect) after single-task or dual-task training. They found better performance for dual-task training compared to single-task training. Based on these findings, they interpret this beneficial practice effect of dual-task training as task-integration by assuming that participants rely their dual-task performance on a conjoint response selection process (Ruthruff et al., 2006) or that it enhances the task-scheduling processes (Strobach et al., 2014).

A second indicator for task-integration can be observed when changing the task order from trial *n*-1 to trial n (De Jong, 1995; Kübler et al., 2022; Luria & Meiran, 2006). Such task-order changes come along with prolonged response times in trial *n*. These findings suggest that the participants seem to represent not only the two tasks of a dual-tasking trial, but also their particular task order.

Our understanding of task integration is similar to this second form, but importantly, we focus on the element-level rather than the task-order level. If the elements of both tasks are contingently combined it facilitates dual-tasking. In most dual-tasking experiments, however, the stimuli of the two tasks are randomly combined such that task integration might not help dual-tasking, but rather contributes to interference costs (Ewolds et al., 2021). To emphasize this difference, we will term this kind of integration *across-task integration*.

Two different strands of dual-tasking research point to the participants’ tendency to integrate the elements across the two tasks. The first line of studies combines an implicit sequence learning task with a second task in which stimuli and responses follow a random rather than a predictable sequence (Schmidtke & Heuer, 1997; Schumacher & Schwarb, 2009). In most of these experiments, the serial reaction time task (SRTT) had been used. In the SRTT, first introduced by Nissen and Bullemer (1987), the participants see four marked locations on the screen which are mapped to respective response keys. In each trial, a stimulus appears at one location on the screen and the participants have to press the assigned response key. Unbeknownst to the participants, the marked screen locations follow a regular and repeating sequence. Implicit learning is a generally robust phenomenon (e.g., Reber, 1993). For instance, the meta-analysis of Oliveira et al. (2023) found that reliability of the SRTT was not significantly affected by factors such as participants’ age, sequence type and variant of computing the index of procedural learning. Yet, sequence learning is often found to be hampered in dual-tasking (Halvorson et al., 2013; Hsiao & Reber, 2001; Röttger et al., 2019, 2021; Schmidtke & Heuer, 1997; Schumacher & Schwarb, 2009). To account for the hampered implicit learning effects in dual-tasking, Schmidtke and Heuer (1997) suspected that the stimuli of both tasks are associated. Participants learn a sequence encompassing a sequence element of the SRTT followed by a random element of the second task. Thus, the random elements of the second task then interrupt the learning of the predictable sequence within the SRTT. To test this assumption more directly, Röttger et al. (2019) trained the participants with a SRTT and a tone-discrimination task. Stimuli of both tasks were always presented concurrently. Importantly, half of the SRTT locations were consistently paired with one specific tone while for the other half, the relation between the SRTT-location and the tones varied randomly. The results revealed that implicit learning was preserved for the fixed, but hampered for the variable SRTT-tone combinations.^1^ This suggests that the stimuli of both tasks within trials had to be associated, before the participants then could account for the sequential predictability across trials. Thus, at least when implicit learning under dual-tasking is considered, the findings seem to point to the assumption that participants integrate the elements of both tasks within each trial (for further evidence see Ewold et al., 2021; Röttger et al., 2021).

The second line of research used a different logic to test for across-task integration. The crucial assumption here is that when the participants would associate the elements of the two tasks one should expect to find enhanced performance if the stimulus–response bindings (*S*–*R* bindings) of both tasks repeat from trial *n*−1 to trial n than when only one repeats while the other changes. This logic has been frequently used in research of feature binding in action control and task switching (cf. Frings et al., 2020; Hommel, 1998; Koch et al., 2018). The assumption is that the repeating stimulus or response might lead to an automatic reactivation (or sustaining activation) of the settings from the previous trial. Yet, in case of a partial repetition (one task repeats, while the other changes) this echo from trial *n*−1 might interfere with the processing of the current trial.

Pelzer et al. (2021) used this logic and observed approximately 50 ms shorter response times when both *S*–*R* bindings repeated from trial *n*−1 to trial n than when only one *S*–*R* binding repeated.^2^ These costs were robust over multiple blocks of practice. The authors assumed that the participants associate the elements of the two tasks and store them together in one single memory episode (Logan, 1988). To further test this assumption, in a second experiment they consistently paired the stimuli of the two tasks. Here, the impact of trial *n*−1 on RT disappeared across practice. Instead, the participants learned the contingencies between the two tasks over training which then superseded the after-effect of the preceding trial. The modulating influence of learning on the impact of trial *n*−1 on RT hence suggests that episodes stored in long-term memory are involved. Additional evidence for the storage of such episodes encompassing the stimulus–response pairings of both tasks in a dual-task trial stem from Zhao et al. (2020). They reported across-task integration effects with a lag of four trials (i.e., three intervening trials). Taken together, the findings of these studies suggest that the participants in dual-tasking experiments do indeed store the elements of both tasks in a conjoint memory episode.

In a second study, Pelzer et al. (2022) trained participants with two tasks, only one of which had a fixed *S*–*R* binding.^3^ The rationale for this manipulation was that a variably assigned target stimulus could not elicit a specific response. If, however, the consideration is correct that the *S*–*R* bindings of both tasks are stored conjointly in one single memory episode, the re-occurrence of the fixed *S*–*R* binding should suffice to re-activate the entire episode (Frings et al., 2020), in particular, the responses to both tasks. The findings revealed evidence for across-task integration effects. Additionally, in another condition in which both tasks contained variable *S*–*R* bindings, no across-task integration effects were obtained. Thus, it seems as if the fixed *S*–*R* binding of at least one task is needed to retrieve both tasks’ responses.

Overall, the reported findings of Pelzer et al., (2021, 2022) suggest that the elements of the two tasks in dual-tasking experiments are associated across tasks and are stored conjointly, meaning that the two tasks are not represented as isolated task-sets. This might be a rather rational consequence of the instruction in dual-tasking experiments (Gozli et al. 2019; Halvorson et al., 2013). Usually, the participants are told to perform the two tasks within one trial, both requiring a response. The trial ends only after having completed both tasks before the next trial starts (Dreisbach, 2012). It is thus conceivable that storing conjoint memory episodes is simply the consequence of presenting the two tasks within one single trial, as is, for example, proposed for automatization of single tasks (Logan, 1988; Logan & Etherton, 1994). As an alternative, however, it might be that storing the two S-R bindings into one single conjoint memory episode presupposes that the stimuli of both tasks appear in temporal proximity. Consequently, if, for instance, the two stimuli are temporally separated by relatively long SOAs, the participants might adopt a more separate representation of the two tasks (Miller et al., 2009; Schumacher & Schwarb, 2009) which then reduces or even eliminates the tendency to associate the task elements across tasks.

In line with this perspective, Schumacher and Schwarb (2009) have shown that implicit sequence learning in dual-tasking is preserved when the two task stimuli are separated by long stimulus-onset asynchronies (SOA) of 800 ms, but hampered when the SOA was set to zero. Taking the reduced implicit learning effect with short SOAs as an indicator for across-task integration and the preserved learning effect as an indicator for a lack of across-task integration (cf. Röttger et al., 2021), this would suggest that long SOAs reduce the tendency to associate the elements across tasks and to store them as conjoint memory episodes. However, Schumacher and Schwarb (2009) only manipulated the long or short SOAs between participants, leading to diverging interpretations. On the one hand, it is conceivable that the long SOAs prevent participants' tendency to store the elements of the two tasks in a conjoint memory episode by provoking a separate representation of the two tasks. On the other hand, storage of conjoint memory episodes may occur as an inevitable consequence of presenting the two tasks within a single trial (Logan, 1988), regardless of SOA length. A long SOA may only reduce the influence of the retrieved memory episodes on task processing in the current trial (Koob et al., 2021).

### The present study

The current experiments aimed to better understand the mechanisms underlying across-task integration in dual tasking. According to the findings of Schumacher and Schwarb (2009), one possibility to hinder such integration of the two tasks’ elements seems to be to separate the presentation of the two stimuli by long SOAs. However, presenting the two stimuli with only long SOAs does not elucidate how exactly this happens. Therefore, we built in the current study on an experimental set-up of Mattes et al. (2021) and Miller et al. (2009) who manipulated the participants’ temporal expectancies by manipulating the frequencies of short and long SOAs (Jentzsch & Sommer, 2002; Miller et al., 2009). The participants received either frequently short SOAs (short frequent condition, SF), or frequently long SOAs (long frequent condition, LF) that were intermixed in both conditions with few medium and few long or short SOAs, respectively.

On the one hand, presenting many long SOAs may reduce the tendency to store the elements of the two tasks within one single memory episode, because it provokes a more separate representation of the two tasks. If this were the case, the across-task integration effects at all SOA levels in the LF condition should be smaller than those in the SF condition. On the other hand, long SOAs may affect the retrieval of the memory episodes. If the stimuli are separated by a long SOA, the retrieval of the conjoint memory episode might be weakened. This, in turn, reduces its influence on task processing in the current trial (Koob et al., 2021). If the latter were the case, we should find across-task integration effects at short, but probably not at long SOAs irrespectively of whether the participants receive many short or many long SOAs during training.

We conducted two experiments with the design of Pelzer et al., (2022, Experiment 1), and manipulated the SOA distributions between participants. The second experiment was a conceptual replication of the first one with slight changes to more precisely test the findings of Experiment 1.

## Experiment 1

The main question targeted in Experiment 1 was if frequently long SOAs can reduce the tendency to associate the task elements by representing the two tasks as separate task-sets. Therefore, in every block the participants received 80% trials with either short SOAs (SF condition) or long SOAs (LF condition) intermixed with 10% trials with medium and long SOAs (SF condition) or 10% medium and short SOAs (LF condition). Based on the findings of Miller et al. (2009), the SF condition should increase the likelihood to represent the two tasks within a single task-set, while the LF condition should induce a more separate task representation. Thus, if the association of task-elements depends on how the two tasks are represented, we should find larger across-task integration effects in the SF condition. If, alternatively, the generation of conjoint memory episodes is an inevitable consequence of presenting two tasks within a single trial, we should find across-task integration effects in both conditions at least with short SOAs.

## Methods

### Participants

Forty-five German speaking participants (12 men, 33 women, mean age years = 23.1 SD = 5.6) were recruited using the crowd sourcing platform Prolific (Palan & Schitter, 2018) and were tested online. They were paid (£6.88) for their participation in the approximately 40-min long online-experiment. An a priori power analysis revealed that a 2 × 3 × 2 × 2 mixed measurement ANOVA with approximately 20 participants would be sensitive to detect large effects of *η*_*p*_^2^ = 0.14 with 90% power (*α* = 0.05).

### Apparatus and stimuli

The experiments were controlled by custom-written software (implemented in PsychoPy v3.0, Peirce et al., 2019). The two tasks consisted of a visual-manual search task (VST) and an auditory-manual discrimination task (ADT) (Pelzer et al., 2022; Experiment 1).

**Fig. 1:**
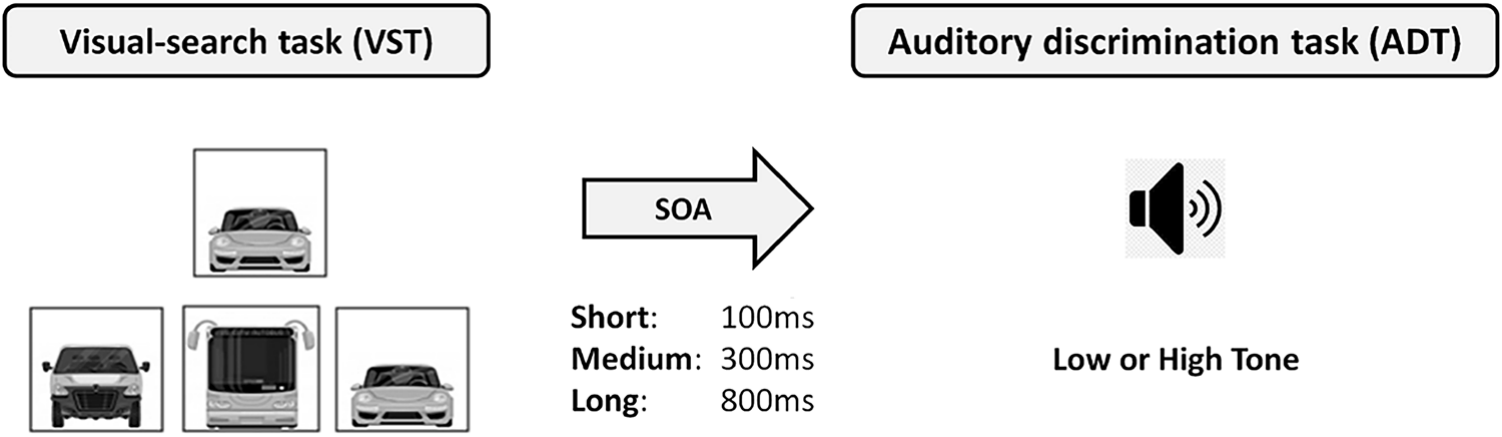
The set-up of one trial. Note that, whenever the VST-target image repeated or switched from trial *n*−1 to trial n, the required response also repeated or switched accordingly

In the VST (Fig. 1), three different car images were presented on three horizontally aligned positions of the screen (100 × 100 pixels, separated by gaps of 300 pixels). The three positions were assigned to spatially mapped response keys (A, S, D on a German QWERTZ keyboard for the left, middle and right position, respectively). A target image (100 × 100 pixels) depicting one of the three car images, appeared 200 pixels above the middle position. Participants were instructed to respond as fast and as accurately as possible with the key aligned to the location of the car image representing the target image. Importantly, analogous to tasks with a fixed mapping between stimulus and response, if the respective target image of the VST repeated or switched from trial *n*−1 to trial n, the location of the response repeated or switched accordingly. On one hand, this way lag 1 effects could be tested. On the other hand, learning of fixed *S*–*R* mappings in the VST was not possible, as the location of the response-pictures otherwise varied across trials.

In the ADT, the participants had to respond to either a high (900 Hz) or a low-pitched tone (300 Hz) which sounded for 56 ms. If they heard a low tone, they were instructed to press the K-key on a German QWERTZ keyboard, for the high tone they should press the L-key (see, Fig. 1). We used in both conditions three different SOAs (100 ms, 300 ms, and 800 ms).

### Procedure

All participants were instructed step by step. They started with 20 practice trials with only the VST and another 20 practice trials with only the ADT. In the last practice block, they received 20 trials of the dual-task. Immediately after this practice phase, the participants performed 6 dual-task blocks of 108 trials each. The stimulus combinations within blocks were presented randomly. The only constraint was that across trials the percentage of full repetitions (the *S*–*R* bindings of both tasks are repeated from trial *n*−1 to trial *n*), partial repetitions of the ADT *S*–*R* binding (only that of the ADT *S*–*R* binding is repeated from trial *n*−1 to trial *n*), partial repetition of the VST *S*–*R* binding (only the VST *S*–*R* binding is repeated), or full switches (the *S*–*R* bindings of both tasks change) were identical within the three SOA levels.

A dual-task trial always began with the presentation of the three different car images at the three positions on the screen. After 200 ms, the VST target (one of the three car images) appeared. After the either short, medium or long SOA (100, 300 or 800 ms, respectively), the auditory stimulus followed. Participants had to locate the position of the target car in the VST and to indicate whether they heard a high or low tone in the ADT by pressing the respective keys assigned to the two tasks.

By instruction, the participants were encouraged to first respond to the VST and then to the ADT but to give both tasks equal priority (Schumacher & Schwarb, 2009). They also were told to respond as fast and as accurately as possible in both tasks. The response-window was always closed after the responses to both tasks had been entered or 2200 ms had passed. An error feedback was not provided at the end of a trial. The next trial started after a fixed inter-trial interval of 750 ms.

### Data analysis

Our main goal was to investigate how the distribution of SOAs of different length will affect the generation of conjoint episodes. If the participants integrate the elements of both tasks, they should be faster if both *S*–*R* bindings repeat between consecutive trials (full repetition) than when only one *S*–*R* binding repeats while the respective other changes (partial repetition). Therefore, we constrained our main analyses to the effects of SOA distribution and SOA-length on these across-task integration effects and categorized the trials with regard to repetitions or changes of the S-R bindings within the VST and the ADT tasks from trial *n*−1 to trial *n*.

We separately conducted for the VST and the ADT 2 (SOA distribution: SF vs. LF) × 3 (SOA-length: 100 ms, 300 ms vs. 800 ms) × 2 (VST-sequence: switch vs. repetition of *S*–*R* binding) × 2 (ADT-sequence: switch vs. repetition of *S*–*R* binding) mixed measurement ANOVAs with either reaction times (RT_VST_ and RT_ADT_) or error rates (ER_VST_ and ER_ADT_) as the dependent variables. The corresponding statistics are included in tables and are not presented in the text**.** Because for the mixed measurement ANOVAs the four-way interaction between ADT sequence, VST sequence, SOA-length, and SOA distribution is of major importance, we further specified this interaction by computing the difference between the mean RT of the partial repetition trials and the mean RT of the full repetition and full switch trials. For the purpose of clarity, we refer to the effects in this specific additional analysis as *across-task binding effects*. In addition, we conducted planned interaction contrasts (SOA distribution × VST sequence × ADT sequence) for each of the three SOA levels to test more precisely for the performance differences between the SF and the LF conditions.

In addition, we analyzed the inter-response intervals (IRIs, the difference between RT_ADT_ and RT_VST_ minus SOA). Based on the findings of, for instance, Ulrich and Miller (2008) and Miller and Ulrich (2008), one could expect that participants tend to group their responses of the two tasks and that this response grouping is more pronounced in the SF than in the LF condition. Response grouping means that participants withhold their first response until the second stimulus has been processed leading to very short IRIs of 100 ms or less. For this purpose, we conducted a 2 (SOA distribution: SF vs. LF) × 3 (SOA-length: 100 ms, 300 ms vs. 800 ms) mixed measurement ANOVA with IRIs as dependent variable.

In all RT-analyses, we excluded trials in which an error had occurred in the VST or the ADT, or in which the RTs were faster than 200 ms or slower than 2000 ms. In the error-analyses, only the trials with too short or too long RTs were excluded. In addition, the first trial of each block was eliminated since it had no precursor. The data-set of participants who made more than 30% errors were excluded from the analyses.

## Results

Overall, the data-set of two participants in the LF condition and that of five participants in SF condition were excluded due to more than 30% incorrect responses in the VST or the ADT. In the LF condition 12.14% and in the SF condition 11.42% of the trials were excluded due to an error or as an RT outlier in the VST or the ADT. We first report the findings with regard to latencies in the two tasks and then with regard to error rates.

### Latencies in the VST and the ADT

**Fig. 2:**
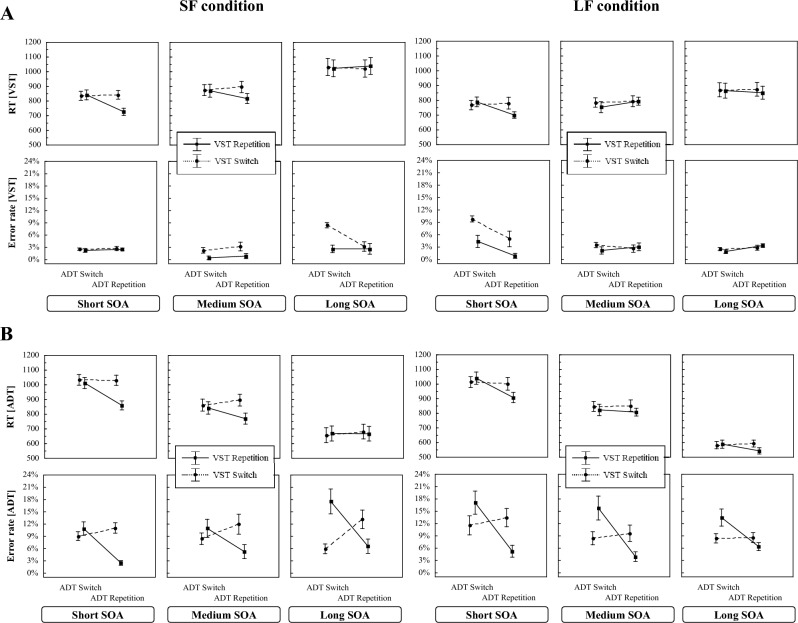
Mean RTs and error rates in the VST (**A**) and the ADT (**B**) in Experiment 1 as a function of SOA distribution, SOA length, the VST sequence and the ADT-sequence. Error bars represent one standard error

Figure 2 depicts in the upper panel the RT_VST_ and the ER_VST_ (Fig. 2A) and in the lower panel the RT_ADT_ and ER_ADT_ (Fig. 2B), both as a function of SOA distribution (SF vs. LF), SOA-length, VST sequence, and ADT sequence, respectively.

#### VST (Task 1)

The 2 × 3 × 2 × 2 mixed measurement ANOVA with RT_VST_ as dependent variable (see Table 1) revealed a significant main effect of SOA-length which was qualified by an interaction between SOA-length and SOA distribution. As can be seen from Fig. 2A, the RT_VST_ increased with longer SOAs and this increase was steeper in the SF than in the LF condition. This suggests that the participants in the two conditions had adopted different processing strategies. In addition, the main effects of the ADT- and the VST-sequences were significant indicating that the participants responded slower when the S-R binding in the ADT or in the VST changed than when both repeated from trial *n*−1 to trial n. Both the ADT- and the VST-sequences interacted with SOA-length. These latter interactions reflect the vanishing impact of the repeating and changing *S*–*R* bindings with increasing SOA.

**Table 1:** Results of the 2 × 3 × 2 × 2 mixed measurement ANOVA for the RTs and error rates in the VST in Experiment 1

Effect	RTs					Error rates		
	*df*_num_	*df*_den_	*F*	*p*	*η*_*p*_^*2*^	*F*	*p*	*η*_*p*_^*2*^
SOA distribution	1	36	3.59	0.066	0.09	1.91	0.175	0.05
SOA length	2	72	44.03	< 0.001	0.55	5.72	< 0.01	0.14
SOA l. × SOA distr	2	72	5.05	< 0.001	0.12	9.92	< 0.001	0.22
VST sequence	1	36	17.99	< 0.001	0.33	40.87	< 0.001	0.53
VST seq. × SOA distr	1	36	0.81	0.375	0.02	0.03	0.859	0.00
ADT sequence	1	36	5.63	0.023	0.14	8.92	< 0.01	0.20
ADT seq. × SOA distr	1	36	1.84	0.184	0.05	0.90	0.349	0.02
SOA l. × VST	2	72	5.50	< 0.01	0.13	1.50	0.230	0.04
SOA l. × VST × SOA distr	2	72	2.60	0.081	0.07	14.90	< 0.001	0.29
SOA l. × ADT	2	72	13.25	< 0.001	0.27	4.11	0.021	0.10
SOA l. × ADT seq. × SOA distr	2	72	2.55	0.085	0.07	11.70	< 0.001	0.25
VST seq. × ADT seq	1	36	13.82	< 0.001	0.28	4.46	0.042	0.11
VST seq. × ADT seq. × SOA distr	1	36	1.39	0.247	0.04	0.02	0.089	0.00
SOA l. × VST seq. × ADT seq	2	72	17.68	< 0.001	0.33	1.58	0.021	0.04
SOA l. × VST seq. × ADT seq. × SOA distr	2	72	6.77	< 0.01	0.16	2.20	0.012	0.06

Important for the question of across-task integration effects, the two-way interaction between ADT and VST sequences was also significant. Figure 2A shows that in both the SF and the LF conditions the repetition of the S-R bindings in both tasks led to faster responses than when they repeated in the VST, but changed in the ADT. This interaction was qualified by a three-way interaction between ADT sequence, VST sequence and SOA-length indicating that the VST x ADT sequence interaction diminished with prolonged SOA.

**Fig. 3:**
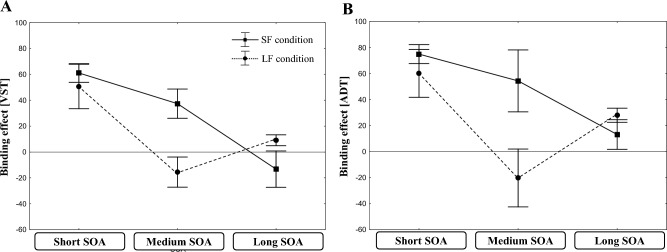
The across-task binding effect (Mean RT of the partial repetition trials minus mean RT of the full repetition and full switch trials) in Experiment 1 for the RTs in the VST (**A**) and the ADT (**B**) as a function of SOA length. Error bars represent one standard error

For the question whether frequently long SOAs can reduce the tendency to associate the task elements by representing the two tasks as separate task-sets, the four-way interaction is of major interest. This interaction was significant. To further specify this four-way interaction between ADT sequence, VST sequence, SOA-length, and SOA distribution, Fig. 3A shows the across-task binding effects for the RT_VST_ as function of SOA-length and SOA distribution. As can be seen from Fig. 3A, with short SOAs, the participants in the LF condition produced large across-task binding effects that were rather of the same size as that in the SF condition. However, at medium SOA, this ADT × VST sequence interaction disappeared for the LF condition, but remained substantial in the SF condition. At long SOAs, both conditions did not show any effect.

These differences between the SF and the LF conditions were further confirmed by separate planned interaction contrasts for either only the medium SOA or the other two SOA-levels (SOA distribution x VST sequence x ADT sequence). For the medium SOA, this contrast was significant (*F*(1,36) = 10.66, *p* < 0.001, *η*_*p*_^*2*^ = 0.23). In contrast, for the short (*F*(1,36) = 0.31, *p* = 0.579, *η*_*p*_^*2*^ = 0.01) and the long (*F*(1,36) = 2.32, *p* = 0.137, *η*_*p*_^*2*^ = 0.06) SOA levels, the interaction contrasts were insignificant.

Thus, the findings revealed that the across-task integration effects at short SOAs occurred independent of the respective SOA distributions. This suggests that a training with frequent long SOAs did not hinder the participants to store the two tasks’ elements in a conjoint memory episode. However, the differences at the medium SOA level suggest that the SOA distribution modulated the effect of these conjoint memory episodes on task performance.

#### ADT (Task 2)

The findings for the RT_ADT_ as dependent variable are shown in Fig. 2B. The 2 × 3 × 2 × 2 mixed measurement ANOVA (see Table 2) mirrored the picture of results for the RT_VST_. The main difference was that, as expected in a PRP-paradigm, the RT_ADT_ decreased significantly with prolonged SOAs. As indicated by a significant interaction between SOA distribution and SOA length, this decrease was significantly steeper in the LF than in the SF condition. This again hints to differences in the processing strategies of the participants according to their respective SOA distribution. In addition, the ANOVA yielded significant main effects of ADT sequence and of VST sequence suggesting that the responses to repetitions of the respective S-R binding from trial *n*−1 to trial n were slightly faster than that for changes. Both main effects did not differ between the SF and LF conditions. The VST sequence interacted with SOA. This interaction was additionally qualified by a three-way interaction between VST-sequence, SOA-length and SOA distribution. The RT_ADT_ difference between changes and repetitions of the S-R binding in the VST was larger at short than at long SOAs and this difference was more pronounced in the SF compared to the LF condition.

**Table 2:** Results of the 2 × 3 × 2 × 2 mixed measurement ANOVA for the RTs and error rates in the ADT in Experiment 1

Effect	RTs					Error rates		
	*df*_num_	*df*_den_	*F*	*p*	*η*_*p*_^*2*^	*F*	*p*	*η*_*p*_^*2*^
SOA distribution	1	36	0.44	0.511	0.01	0.29	0.593	0.01
SOA length	2	72	244.5	< 0.001	0.87	0.51	0.604	0.01
SOA l. × SOA distr	2	72	5.04	< 0.001	0.12	4.28	0.018	0.11
VST sequence	1	36	37.92	< 0.001	0.51	0.30	0.586	0.01
VST seq. × SOA distr	1	36	3.71	0.062	0.09	1.12	0.297	0.03
ADT sequence	1	36	14.14	< 0.001	0.28	16.95	< 0.001	0.32
ADT seq. × SOA distr	1	36	0.04	0.841	0.00	2.46	0.125	0.06
SOA l. × VST	2	72	10.89	< 0.001	0.23	3.18	0.048	0.08
SOA l. × VST × SOA distr	2	72	6.31	< 0.01	0.15	0.74	0.480	0.02
SOA l. × ADT	2	72	18.49	< 0.001	0.34	0.41	0.664	0.01
SOA l. × ADT seq. x SOA distr	2	72	1.60	0.208	0.04	0.44	0.648	0.01
VST seq. × ADT seq	1	36	37.16	< 0.001	0.51	53.63	< 0.001	0.60
VST seq. × ADT seq. × SOA distr	1	36	1.21	0.278	0.03	0.15	0.704	0.00
SOA l. × VST seq. × ADT seq	2	72	10.08	< 0.001	0.22	0.13	0.878	0.00
SOA l. × VST seq. × ADT seq. × SOA distr	2	72	3.54	0.034	0.09	3.81	0.027	0.10

More important, the critical interaction between ADT- and VST-sequences was significant. It was again modulated by SOA length. Thus, also in the ADT, a repetition of the S-R binding leads to faster responses when the S-R binding in the VST repeated from trial *n*−1 to trial n than when the latter changed. This interaction vanished with longer SOAs.

With regard to the important four-way interaction, this also was significant. It is specified in Fig. 3B, which depicts the across-task binding effects for the RT_ADT_ as a function of SOA length and SOA distribution. Again, the two SOA-distribution conditions showed effects at the shortest SOA level. At the medium SOA-level, these effects remained in the SF-condition, but diminished in the LF-condition. The planned three-way interaction contrasts confirmed this impression. As for the VST analyses, the planned three-way interaction contrast (SOA distribution x ADT sequence x VST sequence) for the medium SOA was again significant (*F*(1,36) = 4.23, *p* < 0.05, *η*_*p*_^*2*^ = 0.11), while it was not for the short (*F*(1,36) = 0.56, *p* = 0.458, *η*_*p*_^*2*^ = 0.02) and for the long SOAs (*F*(1,36) = 1.379, *p* = 0.248, *η*_*p*_^*2*^ = 0.04). Consistent with the RT_VST_ results, the findings suggest that frequent long SOAs did not prevent participants from associating the elements of both tasks. At the same time, however, they modulated the effect of these conjoint memory episodes on performance at the intermediate SOA level.

### Error rates in the VST and the ADT

Figure 2 presents the ER_VST_ (Fig. 2A) and the ER_ADT_ (Fig. 2B) as a function of SOA distribution, SOA-length, VST sequence, and ADT sequence.

#### VST (Task 1)

The ER_VST_ was overall relatively low. The 2 × 3 × 2 × 2 mixed measurement ANOVA (see Table 1) yielded a significant main effect of SOA-length which interacted with SOA distribution. In the SF condition, the error rate increased the longer the SOA was, while they decreased in the LF condition. Probably, this reflects the frequency manipulation with a higher error rate for the less frequent SOA-levels. Furthermore, the main effects of VST sequence and ADT sequence were both significant. The participants made more errors when the *S*–*R* bindings of either the VST or the ADT changed than when they repeated. The two-way interaction between SOA-length and VST sequence was not significant, but the three-way interaction was. The SOA-length interacted with ADT sequence and also with SOA distribution. As mentioned already for the SOA effect, we surmise that these latter interactions are also a side effect of the frequency manipulation of the SOAs. With regard to the across-task integration effects, only the VST x ADT sequence interaction was significant.

#### ADT (Task 2)

The ER_ADT_ is overall much higher than the ER_VST_. The analogous ANOVA (see Table 2) showed a significant interaction between SOA-length and SOA distribution. In addition, the main effect of ADT-sequence was significant. The two-way interaction between SOA-length and VST-sequence just reached the level of significance. Regarding the across-task integration effects, the VST x ADT-sequence interaction was significant. The ER_ADT_ in Fig. 2B suggests that the participants made more errors when only one of the two S-R bindings in a trial changed than when both repeated or both changed. This interaction was modulated by SOA distribution and SOA-length. In the SF condition, the size of the VST- x ADT-sequence interaction increased the longer the SOA was, while it decreased in the LF condition.

### Inter-response intervals

To better understand why we found the differences in performance at the medium SOA level even though the frequent long SOAs in the LF condition seemingly did not prevent the participants from associating the elements of both tasks, we analyzed the inter-response intervals. As described in the Data Analysis section, the IRIs should reflect potential differences in the respective processing strategies by higher rates of trials with response grouping in the SF than in the LF condition.

The 2 (SOA distribution) × 3 (SOA length) mixed measurement ANOVA with IRI as the dependent variable confirmed this assumption. The IRIs increased with longer SOAs (*F* (2, 72) = 56.92, *p* < 0.001, *η*_*p*_^*2*^ = 0.613; SF condition: *M*_short_ = 280.37, *M*_medium_ = 282.19, *M*_long_ = 436.37; LF condition: *M*_short_ = 352.66, *M*_medium_ = 354.33, *M*_long_ = 511.91) and, more important, were indeed shorter in the SF than in the LF conditions (*F* (1, 36) = 4.34, *p* = 0.044, *η*_*p*_^*2*^ = 0.11). The interaction was not significant (*F* (1, 36) = 0.24, *p* = 0.787, *η*_*p*_^*2*^ = 0.01). However, even in the SF condition, the IRIs were quite long (larger than 100 ms) which does not really fit with the assumption of response grouping.

## Discussion

In Experiment 1, we found in both SOA conditions substantial across-task integration effects at the shortest SOA level which disappeared with increasing SOA. In addition, the results also revealed a significant influence of the respective SOA distributions. The participants in the LF condition showed almost no across-task integration effects at the medium SOA, while they were still large in the SF condition. Thus, the SOA distribution modulated the across-task integration effects, but the frequently long SOAs in the LF condition did not seem to have prevented the participants from associating the elements of the two tasks. One possibility is that the SOA distribution influenced the participants’ tendency to group their responses (Miller & Ulrich, 2008). In line with this assumption, the IRIs were shorter in the SF condition than in the LF condition. However, even at the shortest SOA level were the IRIs longer than 100 ms which does not support that participants in the SF condition had withhold their first response until they had processed the second stimuli.

Furthermore, a side effect of our manipulation concerning the frequency distribution is that at the medium SOA level the transitions from trial *n*−1 to trial n differed between the two conditions. In the SF condition, the medium SOA was most often preceded by a short SOA, whereas in the LF condition it was a long SOA. Assuming that only the short SOA led to conjoint memory episodes while the long SOA did not, the stronger across-task integration at the medium SOA in the SF condition could simply reflect this difference in the kind of transitions. Experiment 2 served to clarify this latter point.

## Experiment 2

The results of Experiment 1 were somewhat ambiguous about the effect of SOA distribution on the across-task integration effects. Therefore, we repeated Experiment 1 with two modifications. The frequencies of SOA levels (80% short/long, 10% medium, and 10% long/short) were changed into 60% short/long, 30% medium, and 10% long/short SOAs per block in the SF and the LF conditions, respectively. We raised the rate of trials with medium SOAs because they yielded the largest differences between the two conditions. Furthermore, we included a test-phase after the six blocks of training in which the participants of both conditions received identical frequencies of the three SOA levels. The goal of this test-phase was to disentangle the above mentioned confound underlying the large difference in across-task integration effects between the SF and the LF conditions at the medium SOA level. On one hand, it could have been caused by the side effect that, due to our manipulation of the SOA distributions, the frequencies of SOA transitions in successive trials (short to medium vs. long to medium SOA level) differed between the two conditions. In this case, the across-task integration effects should depend on the length of the SOA in the previous trial. On the other hand, however, the found difference in the across-task integration effects at the medium SOA level could reflect different processing strategies in the SF and the LF condition, for instance, a higher rate of response grouping (Miller & Ulrich, 2008). The shorter IRIs in the SF condition as compared to the LF condition of Experiment 1 already seem to support this. If this were indeed the case, the across-task integration effects should be unaffected by the length of the SOA in the preceding trial. Furthermore, if the different processing strategies were temporally stable, the performance in the SF and the LF conditions should differ even when identical frequencies of SOA levels are provided.

Thus, Experiment 2 should help to answer two questions: First, could we replicate the findings of Experiment 1 when the SOA distributions differed less extremely between conditions? Second, were the performance differences between the SF and LF conditions at the medium SOA level in Experiment 1 due to the differences in the SOA transitions or caused by adopting different processing strategies?

## Methods

### Participants

Sixty-three German speaking participants (29 men, 34 women, mean age years = 25.6 SD = 6.5) were recruited using the crowd sourcing platform Prolific (Palan & Schitter, 2018) and tested online. For the participation in the approximately 55-min long online-experiment, the participants received monetary compensation (£7.50). An a priori power analysis revealed that a 2 × 3 × 2 × 2 mixed measurement ANOVA with approximately 20 participants would be sensitive to detect large effects of *η*_*p*_^2^ = 0.14 with 90% power (*α* = 0.05).

### Apparatus, stimuli, and procedure

Apparatus, stimuli, and the procedure were the same as in Experiment 1 with two exceptions. First, we changed the frequency distribution from 80%:10%:10% to 60%:30%:10%, for the trials with short/long, medium, and long/short SOAs in the respective conditions. Second, after six blocks of training, all participants in both the SF and the LF conditions conducted three further blocks in which the SOA-distribution was balanced (30% short, 40% medium and 30% long SOAs). Everything else was identical within these blocks and the participants were not informed about this change.

## Results

Overall, the data-set of seven participants in the LF condition and that of 11 participants in the SF condition were excluded due to more than 30 percent incorrect responses in the VST or the ADT. In the LF condition 10.4% and in the SF condition 11.9% of the trials were excluded due to an error or as an RT outlier in the VST or the ADT. We first report the findings of blocks 1–6 with regard to latencies in the two tasks and then with regard to error rates. Afterwards, we report the results of the test-phase (blocks 7–9).

### Latencies in the VST and the ADT (Blocks 1–6)

**Fig. 4:**
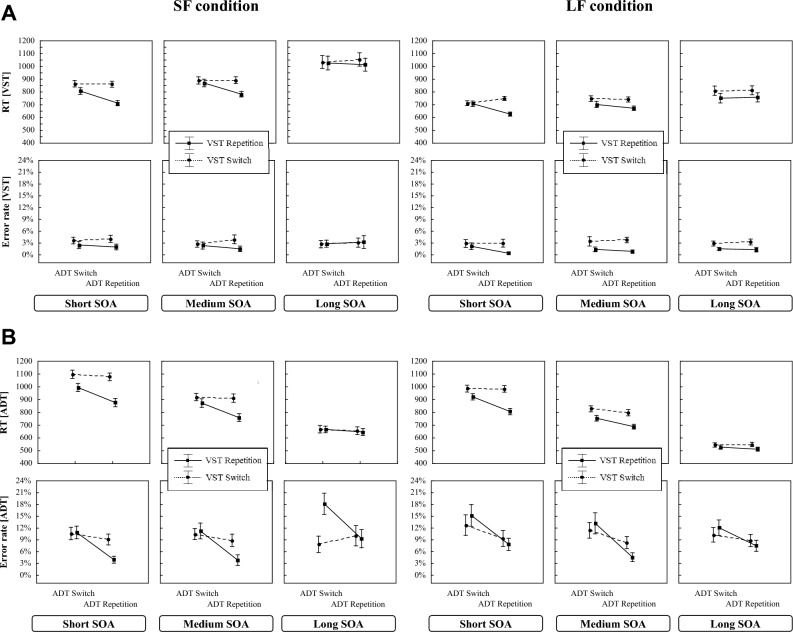
RTs and error rates in the VST (**A**) and the ADT (**B**) in Blocks 1–6 of Experiment 2 as a function of SOA distribution, SOA length, ADT-sequence and VST-sequence. Error bars represent one standard error

Figure 4 shows in the upper panel the RT_VST_ and the ER_VST_ (Fig. 4A) and in the lower panel the RT_ADT_ and ER_ADT_ (Fig. 4B) as a function of SOA distribution, SOA length, VST-sequence, and ADT-sequence, respectively.

#### VST (Task 1)

As can be seen from Fig. 4A, the findings largely resemble those of Experiment 1. The 2 × 3 × 2 × 2 mixed measurement ANOVA (see Table 3) yielded a significant main effect of SOA distribution due to slower responses in the SF condition than in the LF condition. In addition, the main effect of SOA length was also significant and was again qualified by an interaction between SOA length and SOA distribution. Figure 4A shows that the RT_VST_ increases with longer SOAs and this increase was stronger in the SF than in the LF condition (cf. Mattes et al., 2021). Furthermore, the main effects of ADT-sequence and of VST-sequence were significant indicating that the participants responded slower when the S-R binding in the ADT or in the VST changed than when it repeated. Both main effects interacted with SOA suggesting that the RT-differences between repeated and changed *S*–*R* bindings decreased with increasing SOA levels.

**Table 3:** Results of the 2 × 3 × 2 × 2 mixed measurement ANOVA for the RTs and error rates in the VST in Experiment 2

Effect	RTs					Error rates		
	*df*_num_	*df*_den_	*F*	*p*	*η*_*p*_^*2*^	*F*	*p*	*η*_*p*_^*2*^
SOA distribution	1	43	19.41	< 0.001	0.31	0.51	0.479	0.01
SOA length	2	86	43.44	< 0.001	0.50	0.06	0.942	0.00
SOA l. × SOA distr	2	86	8.69	< 0.001	0.17	0.60	0.554	0.01
VST-sequence	1	43	133.7	< 0.001	0.76	21.48	< 0.001	0.33
VST × SOA distr	1	43	0.23	0.635	0.01	2.40	0.128	0.05
ADT-sequence	1	43	27.39	< 0.001	0.40	0.04	0.851	0.00
ADT × SOA distr	1	43	4.00	0.031	0.10	0.79	0.379	0.02
SOA l. × VST	2	86	11.13	< 0.001	0.21	2.66	0.076	0.06
SOA l. × VST x SOA distr	2	86	7.85	< 0.001	0.15	1.40	0.253	0.03
SOA l. × ADT	2	86	13.48	< 0.001	0.24	0.50	0.605	0.01
SOA l. × ADT × SOA distr	2	86	1.45	0.241	0.00	0.09	0.911	0.00
VST × ADT	1	43	56.12	< 0.001	0.57	7.58	< 0.01	0.15
VST × ADT × SOA distr	1	43	2.55	0.117	0.06	0.002	0.878	0.00
SOA l. × VST × ADT	2	86	14.17	< 0.001	0.25	0.67	0.512	0.02
SOA l. × VST × ADT × SOA distr	2	86	3.94	0.023	0.01	0.54	0.586	0.01

More important for our research question, the two-way interaction between ADT- and VST-sequences, indicating across-task integration effects, was also significant. This interaction was qualified by a three-way interaction between ADT-sequence, VST-sequence and SOA length. It was caused by the ADT × VST sequence interaction diminishing with prolonging SOAs.

**Fig. 5:**
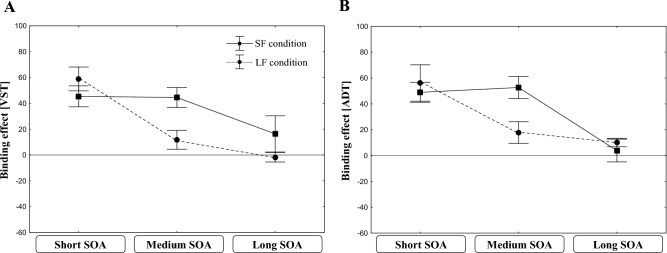
The across-task binding effect (Mean RT of the partial repetition trials minus mean RT of the full repetition and full switch trials) in Experiment 2 for the RTs in the VST (**A**) and the ADT (**B**) as a function of SOA length. Error bars represent one standard error

With respect to the question whether frequently long SOAs can reduce the tendency to associate the task elements by representing the two tasks as separate task-sets, the four-way interaction is the most important. Again, this interaction was significant. Analogous to Experiment 1, we further specified this interaction by analyzing the across-task binding effects for the RT_VST_ as a function of SOA length and SOA distribution. This interaction is depicted in Fig. 5A. Replicating the result pattern of Experiment 1, we found substantial across-task binding effects at the short SOA level for both conditions. In the LF condition, this effect disappears at the medium SOA level, whereas it remains in the SF condition. At the long SOA level, both conditions show almost no effects.

We conducted again separate planned interaction contrasts (SOA distribution x ADT-sequence x VST-sequence) for the three different SOA levels. These confirmed that only the across-task binding effects for the medium SOA level differed significantly between the SF and the LF conditions (*F*(1,43) = 9.57, *p* < 0.01, *η*_*p*_^*2*^ = 0.19). In contrast, neither for the short nor for the long SOAs the interaction contrasts indicated significant differences between conditions (short SOA (*F*(1,43) = 1.20, *p* = 0.280, *η*_*p*_^*2*^ = 0.03; long SOAs (*F*(1,43) = 1.64, *p* = 0.211, *η*_*p*_^*2*^ = 0.04).

Thus, even though we changed the frequencies of the three SOA-levels, the findings of blocks 1–6 replicated those of Experiment 1. The across-task integration effects were influenced by the current length of the respective SOA and also by the frequency distribution of the SOAs.

#### ADT (Task 2)

The RT_ADT_ are depicted in Fig. 4B. Again, the 2 × 3 × 2 × 2 mixed measurement ANOVA revealed a similar picture as the one obtained for the VST (see Table 4). As expected for the second task in a PRP-paradigm, the RT_ADT_ significantly decreased with longer SOAs. In contrast to Experiment 1, this decrease was of comparable size in the LF- and the SF-conditions. As for the RT_VST_, the main effects of the ADT- and the VST-sequences were significant indicating that the participants responded faster when the *S*–*R* binding in the ADT or the VST repeated than when it changed. These RT-differences decreased with longer SOA. Again, only the three-way interaction between SOA distribution, SOA length, and VST-sequence was significant due to a larger difference between repeating and changing *S*–*R* bindings at the short and the medium SOAs in the SF condition.

**Table 4:** Results of the 2 × 3 × 2 × 2 mixed measurement ANOVA for the RTs and error rates in the ADT in Experiment 2

Effect	RTs					Error rates		
	*df*_num_	*df*_den_	*F*	*p*	*η*_*p*_^*2*^	*F*	*p*	*η*_*p*_^*2*^
SOA distribution	1	43	9.90	< 0.01	0.19	0.07	0.797	0.00
SOA length	2	86	801.9	< 0.001	0.95	3.12	0.049	0.07
SOA l. × SOA distr	2	86	2.24	0.112	0.05	6.33	< 0.01	0.13
VST-sequence	1	43	170.2	< 0.001	0.80	0.00	0.995	0.00
VST × SOA distr	1	43	0.23	0.631	0.01	0.03	0.857	0.00
ADT-sequence	1	43	85.94	< 0.001	0.67	39.86	< 0.001	0.48
ADT × SOA distr	1	43	1.50	0.228	0.03	0.28	0.602	0.01
SOA l. × VST	2	86	74.67	< 0.001	0.64	9.19	< 0.001	0.18
SOA l. × VST × SOA distr	2	86	3.60	0.031	0.08	7.29	< 0.01	0.14
SOA l. × ADT	2	86	25.74	< 0.001	0.37	2.09	0.130	0.05
SOA l. × ADT × SOA distr	2	86	0.02	0.979	0.00	0.39	0.676	0.01
VST × ADT	1	43	71.03	< 0.001	0.62	40.48	< 0.001	0.48
VST × ADT × SOA distr	1	43	0.91	0.347	0.02	3.09	0.086	0.07
SOA l. × VST × ADT	2	86	13.09	< 0.001	0.23	1.08	0.344	0.02
SOA l. × VST × ADT × SOA distr	2	86	3.56	0.033	0.08	3.09	0.051	0.07

Concerning the across-task integration effect, we could replicate also the significant interaction between the ADT- and the VST-sequences. Important for the research question, this interaction was again influenced by SOA length and also by SOA-distribution. To specify this four-way interaction, Fig. 5B depicts the across-task binding effects for the RT_ADT_ as a function of SOA length and SOA distribution. The picture is similar to that of the VST. While the two conditions showed large effects at the short SOA level, they diminished at the medium SOA level in the LF, but not in the SF condition. For the long SOAs, no effects were found for both conditions.

The planned three-way interaction contrasts (SOA distribution x ADT-sequence x VST-sequence) confirmed this impression. For the medium SOAs, the contrast was again significant (*F*(1,43) = 8.49, *p* < 0.01, *η*_*p*_^*2*^ = 0.16), whereas it was not for the short (*F*(1,43) = 0.20, *p* = 0.659, *η*_*p*_^*2*^ = 0.00) or long SOAs (*F*(1,43) = 0.45, *p* = 0.506, *η*_*p*_^*2*^ = 0.01).

Thus, the results for both the VST and the ADT replicated those of Experiment 1, indicating that these findings were rather robust and were only slightly affected by changing the SOA distributions for the SF and LF conditions from Experiment 1 to Experiment 2.

### Error rates in the VST and the ADT (Blocks 1–6)

Figure 4A depicts the ER_VST_ and Fig. 4B the ER_ADT_. Both dependent variables are displayed as a function of SOA distribution, SOA length, VST-sequence, and ADT-sequence.

#### VST (Task 1)

As in Experiment 1, error rates in the VST were overall relatively low. Here, the 2 × 3 × 2 × 2 mixed measurement ANOVA (see Table 3) yielded only a significant main effect of VST-sequence which was due to lower error rates when the *S*–*R* binding repeated than when it changed. In addition, the interaction between the VST-sequence and the ADT-sequence was significant, indicating across-task integration effects. In both conditions the participants made fewer errors when the *S*–*R* bindings of both tasks repeated than when only the *S*–*R* binding of the VST repeated, but the *S*–*R* binding in the ADT changed from trial *n*−1 to trial n. This finding mirrors the results of the latencies in the VST.

### ADT (Task 2)

As in Experiment 1, the participants of both conditions made more errors in the ADT than in the VST. The analogous mixed measurement ANOVA (see Table 4) showed a significant main effect of SOA length which differed between the two SOA distributions. This interaction was mainly driven by the higher error rates for long SOAs in the SF condition. In addition, the main effect of ADT-sequence was significant, indicating fewer errors when the *S*–*R* binding in the ADT repeats than when it changed. For the VST-sequence, the ANOVA yielded a significant VST-sequence × SOA-length interaction which was modulated by SOA distribution. Regarding the across-task integration effects, the VST- × ADT-sequence interaction was also significant. The error rates in Fig. 3B show that the participants in both conditions made fewer errors when the *S*–*R* bindings of both tasks repeated than when only the *S*–*R* binding of one task repeated.

### Inter-response intervals (Blocks 1–6)

To further investigate the found performance differences at the medium SOA level, which occurred even though the frequent long SOAs in the LF condition seemingly did not prevent the participants from associating the elements of both tasks, we analyzed the inter-response intervals. Analogous to Experiment 1, the IRIs should reflect potential differences in the respective processing strategies (cf. response grouping) by shorter IRIs in the SF than in the LF condition.

We conducted a 2 (SOA distribution) × 3 (SOA-length) ANOVA with IRIs as dependent variable. The main effect of SOA distribution failed the level of significance (*F*(1, 43) = 2.56, *p* = 0.117, *η*_*p*_^*2*^ = 0.06). However, the main effect of SOA length was significant (*F*(2, 86) = 109.78, *p* < 0.001, *η*_*p*_^*2*^ = 0.72), as was the interaction between SOA length and SOA distribution (*F*(2, 86) = 7.88, *p* < 0.001, *η*_*p*_^*2*^ = 0.155). The IRIs increased the longer the SOAs were and this increase was less steep in the SF condition (*M*_short_ = 302.63, *M*_medium_ = 307.33, *M*_long_ = 430.83) than in the LF condition (*M*_short_ = 324.88, *M*_medium_ = 350.62, *M*_long_ = 553.49). Even though the increase in the SF condition at long SOAs was less steep, the IRIs were quite long in the SF condition which does not fit well with the assumption of response grouping (Ulrich & Miller, 2008).

### Performance in the test phase (Blocks 7–9)

As already mentioned in the introduction to Experiment 2, in Blocks 7 to 9, all participants received the same frequencies of the three SOA levels. This was done to further clarify whether the stronger across-task integration effect found in the SF condition at the medium SOA level was caused by different processing strategies in the SF versus LF conditions or whether the observed effect rather derives from a confound caused by the different frequencies of short to medium and long to medium SOA transitions in the SF compared to the LF condition. If the latter were the case, we should find that the size of the across-task integration effects for the medium SOA trials are influenced by the respective SOA in the preceding trial. Therefore, we limited the presentation of the results on only the trials with a medium SOA and compared the transitions from short to medium and from long to medium SOAs in both conditions.

### Latencies in the VST and the ADT (Blocks 7–9)

**Fig. 6:**
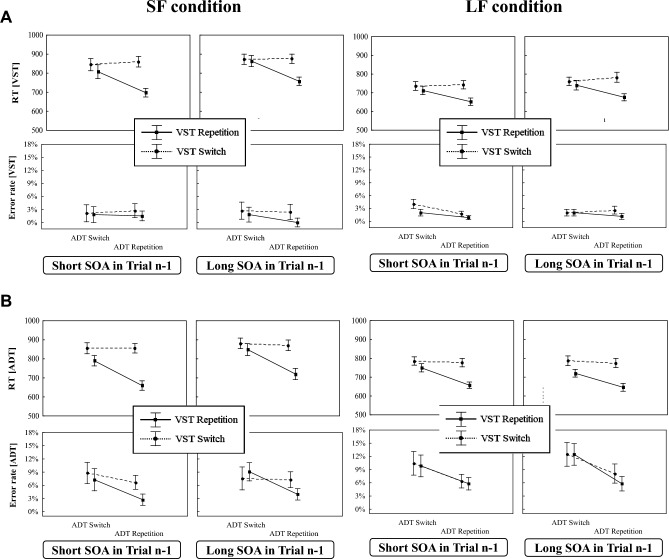
RTs and error rates for the medium SOA trials in the VST (**A**) and the ADT (**B**) in the blocks 7–9 as a function of SOA distribution, SOA transition (long versus short SOA in trial *n*−1), VST-sequence and ADT-sequence. Error bars represent one standard error

Figure 6 depicts in the upper panel the RT_VST_ and ER_VST_ (Fig. 6A) and in the lower panel the RT_ADT_ and ER_ADT_ (Fig. 6B) as a function of SOA distribution, SOA transition, VST-sequence and ADT-sequence for only the medium SOA trials.

#### VST (Task 1)

We conducted a 2 (SOA distribution: SF vs. LF) × 2 (SOA transition: short-medium vs. long-medium) × 2 (VST-sequence: repetition vs. switch) × 2 (ADT-sequence: repetition vs. switch) mixed measurement ANOVA with RT_VST_ as dependent variable (see Table 5). This ANOVA yielded significant main effects of SOA distribution, of SOA transition, of VST-sequence, and of ADT-sequence. As depicted in Fig. 6A, the participants in the SF-condition responded overall slower than those in the LF condition. In both conditions, the RT_VST_ was shorter when the SOA in the preceding trial was short than when it was long, when the *S*–*R* binding in the VST repeated than when it changed, and also when the *S*–*R* binding in the ADT repeated than when it changed. The ADT-sequence effect interacted significantly with the SOA distribution indicating that the difference between the repeated versus changed *S*–*R* bindings in the ADT was larger in the SF condition than in the LF condition. Additionally, the three-way interaction between SOA distribution, SOA transition and VST-sequence was significant. As can be seen in Fig. 6A, the participants in the SF condition responded slower than the participants in the LF condition when the preceding trial contained a short SOA than when it contained a long SOA and this was particularly the case when the S-R binding in the VST changed.

**Table 5:** Results of the 2 × 2 × 2 × 2 mixed measurement ANOVA for the RTs and error rates in the VST in test phase (Blocks 7–9) of Experiment 2

Effect	RTs					Error rates		
	*df*_num_	*df*_den_	*F*	*p*	*η*_*p*_^*2*^	*F*	*p*	*η*_*p*_^*2*^
SOA distribution	1	43	8.30	< 0.01	0.16	0.06	0.802	0.00
SOA transition	1	43	34.41	< 0.001	0.44	0.40	0.530	0.01
SOA trans. × SOA distr	1	43	0.84	0.364	0.02	0.01	0.912	0.00
VST-sequence	1	43	137.8	< 0.001	0.76	14.03	< 0.001	0.25
VST × SOA distr	1	43	3.07	0.087	0.07	0.04	0.802	0.00
ADT-sequence	1	43	49.76	< 0.001	0.54	3.09	0.086	0.07
ADT × SOA distr	1	43	6.03	0.018	0.12	0.38	0.543	0.01
SOA trans. × VST	1	43	3.35	0.074	0.07	0.02	0.890	0.00
SOA trans. × VST × SOA distr	1	43	6.71	0.013	0.14	1.52	0.224	0.03
SOA trans. × ADT	1	43	0.00	0.955	0.00	0.08	0.778	0.00
SOA trans. x ADT x SOA distr	1	43	0.49	0.490	0.01	4.35	0.043	0.09
VST × ADT	1	43	109	< 0.001	0.72	1.05	0.310	0.02
VST × ADT × SOA distr	1	43	4.66	0.036	0.10	0.65	0.424	0.01
SOA trans. × VST × ADT	1	43	0.01	0.933	0.00	1.49	0.228	0.03
SOA trans. × VST × ADT × SOA distr	1	43	1.24	0.272	0.03	0.58	0.451	0.01

The ANOVA also yielded a significant interaction between VST- and ADT-sequences, indicating across-task integration effects. Most importantly, this interaction was qualified by SOA distribution, but not by SOA transition.^4^

Thus, even though the participants in both conditions had received the same distribution of the SOAs in Blocks 7–9, the across-task integration effects differed between conditions. They were more pronounced in the SF than in the LF condition. In addition, the respective transitions from short to medium SOAs or from long to medium SOAs did not affect the size of the across-task integration effect at the medium SOA level, neither in the SF nor in the LF conditions.

#### ADT (Task 2)

Figure 6B depicts the analogous results for the RT_ADT_. The results of the mixed measurement ANOVA are shown in Table 6. As for the RT_VST_, this ANOVA revealed significant main effects of SOA distribution, of SOA transition, of VST-sequence and ADT-sequence. The participants in the SF-condition were overall slower than the participants in the LF condition. In both conditions, the participants responded slower when the SOA in the preceding trial was short than when it was long, when the *S*–*R* binding in the VST changed than when it repeated, and when the *S*–*R* binding in the ADT changed than when it repeated. None of these latter two main effects were modulated by SOA distribution. As for the RT_VST_, there was again a significant three-way interaction between SOA distribution, SOA transition and VST-sequence. Also in the ADT, the participants in the SF condition responded slower than those in the LF condition when the preceding trial contained a short SOA compared to when it contained a long SOA. This was again particularly the case when the *S*–*R* binding in the VST changed.

**Table 6:** Results of the 2 × 2 × 2 × 2 mixed measurement ANOVA for the RTs and error rates in the ADT in test phase (Blocks 7–9) of Experiment 2

Effect	RTs					Error rates		
	*df*_num_	*df*_den_	*F*	*p*	*η*_*p*_^*2*^	*F*	*p*	*η*_*p*_^*2*^
SOA distribution	1	43	5.20	0.028	0.11	1.02	0.319	0.02
SOA transition	1	43	6.82	0.012	0.14	2.45	0.125	0.05
SOA trans. × SOA distr	1	43	18.76	< 0.001	0.30	0.49	0.486	0.01
VST-sequence	1	43	165.4	< 0.001	0.79	2.30	0.137	0.05
VST × SOA distr	1	43	2.31	0.136	0.05	0.29	0.596	0.01
ADT-sequence	1	43	88.52	< 0.001	0.67	16.66	< 0.001	0.28
ADT × SOA distr	1	43	2.91	0.095	0.06	0.84	0.365	0.02
SOA trans. × VST	1	43	1.22	0.275	0.03	0.31	0.578	0.01
SOA trans. × VST × SOA distr	1	43	11.80	< 0.01	0.22	1.18	0.284	0.03
SOA trans. × ADT	1	43	0.04	0.835	0.00	0.10	0.751	0.00
SOA trans. × ADT × SOA distr	1	43	0.55	0.463	0.01	0.61	0.440	0.01
VST x ADT	1	43	85.68	< 0.001	0.67	3.64	0.063	0.08
VST × ADT × SOA distr	1	43	5.62	0.022	0.12	0.97	0.330	0.02
SOA trans. × VST × ADT	1	43	0.59	0.447	0.01	0.60	0.444	0.01
SOA trans. × VST × ADT × SOA distr	1	43	0,04	0.842	0.00	0.00	0.986	0.00

The ANOVA yielded again a significant interaction between VST- and ADT-sequence, indicating an across-task integration effect. More important for the main question of the test phase, this interaction was modulated by SOA distribution, but, as for the RT_VST_, not by SOA transition.^5^

The across-task integration effects for the RT_ADT_ mirror the results of the RT_VST_. They were overall smaller in the LF- than in the SF-condition, but did not differ with respect to the transitions from short to medium versus from long to medium SOA. Thus, we can exclude the alternative explanation that the differences between the SF and LF conditions at the medium SOA levels observed during training were caused by the different frequencies of short to medium and long to medium SOA transitions.

### Error rates in the VST and the ADT (Blocks 7–9)

Figure 4A shows the ER_VST_ and Fig. 4B the ER_ADT_ as a function of SOA distribution, SOA transition, VST-sequence, and ADT-sequence for the medium SOAs. As can be seen in Fig. 4, the error rates in the VST were again lower than that in the ADT.

#### VST (Task 1)

The 2 × 2 × 2 × 2 mixed measurement ANOVA (see Table 5) yielded only a significant main effect of VST-sequence. This was due to larger error rates when the S-R binding in the ADT changed than when it repeated. In addition, the three-way interaction between SOA transition, ADT-sequence, and SOA distribution was significant. The error rates in the SF condition were less affected by the repetition vs. the change of the ADT stimulus when the SOA in trial *n*−1 was long than when it was short. This difference was not present in the LF condition. No other effect reached the level of significance.

#### ADT (Task 2)

The analogous ANOVA for the ADT (see Table 6) only showed a significant main effect of ADT-sequence. In both conditions the error rates were smaller when the *S*–*R* binding in the ADT repeated than when it changed. All other effects failed or just failed the level of significance.

## Discussion

To summarize, the overall pattern of results of Experiment 2 replicated those of Experiment 1 even with less extreme frequency distributions between the two conditions. At the short SOA level, we obtained clear evidence for across-task integration effects in both conditions. This fits more with the assumption that the generation of conjoint memory episodes is an inevitable consequence of presenting the two tasks within one trial (Logan, 1988). In addition, we also found that the across-task integration effects disappeared faster in the LF condition than in the SF condition. While they almost vanished in the LF condition at the medium SOA level, they were still substantial in the SF condition. Importantly, the results of the additional test phase excluded that this performance difference between conditions was caused by the higher frequencies of short to medium SOA transitions in the SF condition compared to the LF condition. Furthermore, the analyses of the IRIs in Blocks 1–6 revealed that these were shorter in the SF condition than in the LF condition. Together these findings suggest that the manipulation of the SOA frequencies did not hinder the participants to associate the elements of the two tasks. Rather, it may have altered the tendency to group the responses of the two tasks. In the SF condition, the frequent short gaps between the two stimuli may have led participants to withhold their response to the first stimulus for a short time. In the LF condition, on the other hand, participants entered their response to the first stimulus immediately after generating it. However, as mentioned above, the IRIs were much longer than expected for response grouping (Ulrich & Miller, 2008).

## General discussion

The current study aimed at testing whether the generation of conjoint memory episodes is an inevitable consequence of presenting two tasks within a single trial (Logan, 1988). Alternatively, it is conceivable that providing frequent long SOAs can lead participants to represent the two tasks separately and as such reduce or even fully prevent participants to generate conjoint memory episodes (Schumacher & Schwarb, 2009). For this purpose, we manipulated the distribution of different SOAs (100 ms, 300 ms, or 800 ms) by presenting either frequent short (SF condition) or frequent long SOAs (LF condition) (Miller et al., 2009). This design made it possible to investigate the effect of SOA on across-task integration on two levels: On one hand, we can test whether a preceding short as compared to a preceding long SOA modulates across-task integration in the current trial. On the other hand, it is possible to investigate if a high frequency of long SOAs reduces across-task integration as it leads to a more separate representation of the two tasks (Schumacher & Schwarb, 2009). This is plausible if one assumes that participants likely register the frequent length of the temporal gap between the presentation of the two stimuli and in turn adapt their processing strategy. With frequent long SOAs, they may tend to enter their responses immediately after generating it. In contrast, experiencing a frequent short temporal gap between the two stimuli might provoke a tendency to postpone entering the response to the first stimulus until the second stimulus had been presented.

Experiments 1 and 2 yielded two important results: First, in both experiments, the SOA distribution influenced the participants’ processing strategies. The RTs in the VST increased with SOA length and this increase was steeper in the SF than in the LF conditions. The ADT reflected the reversed pattern; the RTs decreased with SOA length and this was more pronounced in the LF than in the SF conditions. Furthermore, the IRIs were shorter in the SF condition compared to the LF condition. Together, this pattern of results is consistent with the assumption that the frequency distribution of the SOAs modulated the processing strategy.

Second, the interaction between the VST and the ADT-sequences, as the indicator for across-task integration, was overall significant. The interaction was affected by the length of the SOA. It diminished in both conditions with increasing length of the SOA. Most importantly, for short SOAs, the across-task integration effects were of rather the same size in both conditions, meaning that the frequent long SOAs in the LF condition did not hinder the participants from storing the stimuli of both tasks in one conjoint memory episode. However, at the medium SOA level the across-task integration effects largely differed between the two conditions. While the effects were still large in the SF condition, they almost vanished in the LF condition.

With the test phase, additionally introduced after the training in Experiment 2, we could exclude that this difference in the across-task integration effects at the medium SOA level were caused by differences in the SOA transitions (more short-to-medium transitions in the SF and more long-to-medium transitions in the LF condition). With identical distributions of SOAs in the test phase, both conditions showed substantial across-task integration effects at the medium SOA level. This interaction effect was modulated by the respective SOA distribution during training. Yet, it was not affected by the respective SOA of the preceding trial. Even when a long SOA in trial *n*−1 preceded the medium SOA in trial n, the participants of both conditions produced large across-task integration effects. In combination with the observation that in both experiments, the interaction between the VST and the ADT-sequences was present at the short SOA level irrespective of SOA distribution, this points to the ubiquity of encoding the two tasks’ elements conjointly.

Taken together, the current findings are in line with the assumption that in dual tasking the storage of the elements of both tasks in conjoint memory episodes itself seems to be a rather basic process which does not depend on the length of the SOA or on the respective processing strategy. Rather, it seems to be an inevitable consequence of presenting two tasks within one single trial (Logan, 1988). This fits with former findings from implicit sequence learning in dual-tasking showing that the participants need to associate the two tasks’ elements before they then can exploit the regularity built into the stimulus- and response sequence of one of the two tasks (Röttger et al., 2019, 2021; Schmidtke & Heuer, 1997). Even when the participants kept the two tasks more separate, as was likely the case in the current LF condition, this does not prevent this common storage. However, it seems to help reducing the influence of the reactivated conjoint memory episode. In case of frequent short SOAs, an even longer SOA is needed to overcome the influence of the reactivated memory episode on the current task processing (Koob, 2021; Logan & Gordon, 2001).

However, it is important to point out a potentially problematic aspect of our pattern of results. Our findings concerning across-task integration were solely based on the facilitation effect when both *S*–*R* bindings repeated. We found negligible effects when the *S*–*R* bindings of both tasks changed from trial *n*−1 to trial n. This is at odds, with partial repetition costs usually found in single task experiments of action control (cf. Hommel, 1998, 2006). The assumption underlying these single-task experiments is that the partial repetition costs result from the binding of feature codes in an event file. These feature codes can or cannot overlap across tasks. If an event file already occupies a feature code, the creation of the next event file is delayed leading to costs in the case of a partial repetition. If the two tasks do not overlap in their features (full switch), a new event file is immediately generated resulting in shorter response times. However, in a recently published paper, Koch et al. (2023) argued that alternatively the shorter RTs in case of a full switch could result from expectancy effects. Usually, these paradigms use only two-choice discrimination tasks. This implies that for full switches, participants can detect that nothing repeats and very quickly decide to press the respective other keys for both tasks. The findings of Koch et al. (2023) supported this conjecture. When using four different response alternatives the full switch benefit was substantially reduced. Since we used three response alternatives in our VST such a bias toward pressing the respective other response-key does not work. This might have contributed to the fact that we found benefits for full repetition, but not the full switch of the S-R bindings.

The lack of a full-switch benefit may also suggest reconsidering the interpretation of our across-task integration effects for another reason. While participants benefit from the repetition of both *S*–*R* bindings, it is less clear whether the repetition of only one *S*–*R* binding has a cost in our experiments. Alternatively, one could assume that when both *S*–*R* bindings repeat it leads to the retrieval of the episode from the preceding trial, whereas in trials with only one repeating *S*–*R* binding, no such retrieval occurs at all. According to Hintzman (1990) or Jamieson and Mewhort (2009), one option is to assume that the strength of the echo of the memory episode depends on the similarity between the content of the memory episode and the current stimulus combination. This would imply that if both *S*-*R* bindings repeat from trial *n*−1 to trial *n*, the memory episode leads to a strong echo. If only one *S*-*R* binding repeats while the respective other changes, the echo of the reactivated memory episode is probably too weak to facilitate task processing (Hintzman, 1990; Jamieson & Mewhort, 2009). A too week echo, does not result in the retrieval of the episode and thus in no interference-based costs. The same would be true if both stimuli change from trial *n*−1 to trial *n*.

This also might explain why we found no across-task integration effects at long SOAs. In trials with long SOAs the single stimulus might already activate the respective memory episode, but according to the above consideration, the strength of the echo of the memory episode might be weaker compared to trials with short SOAs. It is also conceivable that the echo of the memory episode fades out rather quickly (Koob et al., 2021; Logan & Gordon, 2001). This would also weaken its effect on the response generation in trials with long SOAs. Though speculative, these considerations might be suited to explain the current findings.

So far, we have argued that the RT-difference between the repetition of both versus only one *S*-*R* binding indicates that both *S*-*R* bindings were stored as a conjoint memory episode. However, as mentioned above, this assumption relies only on the response repetition benefit that drove the obtained interactions between the VST and the ADT-sequences. This raises the question whether alternative assumptions can explain the observed benefit when both *S*-*R* bindings repeat without attributing this effect to the storage of memory episodes.

For instance, Houghton and Hartley (1995) suggest a “repetition mode” to explain how doubling of reactions in typing can be solved which is a severe problem for models of serial order. Based on analysis of typing errors, the authors provide some evidence that such a repetition mode is used. For instance, a common typing error in words with double letters is that the doubling is shifted to a different letter [diiferent leeter], as if “doubling” was something one could switch on and off (see also Mattler, 2005, for a similar proposal).

Transferred to the found interaction between the VST and the ADT-sequences which was based on the strong benefit when both tasks’ elements repeat, this would imply that the participants simply detect that both stimuli are the same as in the preceding trial. The “repetition mode” can thus be used. If only one stimulus repeated, this repetition mode (“just repeat everything”) cannot be used. One could argue that such a view is somewhat different from the encoding of conjoint episodes. All that is needed is to detect whether there is or is no change of elements in the two tasks. The repetition benefit of the two *S*-*R* bindings would then reflect that the repetition mode cannot be easily switched on or off for only one of the two tasks within a trial. Rather, it is effective only when the stimuli of both tasks repeat, which means that, similar to our across-task integration assumption, the participants seem to represent the stimuli of both tasks as belonging together.

Furthermore, repetition effects that span longer than a single trial do not seem to be attributable to such a mechanism. For instance, Zhao et al. (2020) observed episodic effects spanning four trials. Thus, it is unlikely that the current finding could be fully attributed to such a “repetition mode” while leaving nothing for episodic encoding and retrieval. In addition, the findings of Pelzer et al. (2021), already mentioned in the Introduction section, does not fit well with such an explanation. They found that when the participants were trained with contingent pairings of the elements of the two tasks the after-effect of the trial *n*−1 pairing vanished. This suggests that the participants have acquired memory episodes of the contingent task pairings which can supersede the influence of the most recently generated memory episode (in trial *n*−1). Furthermore, also the observations of Röttger et al. (2021) corroborate the assumption of conjoint memory episodes. In a dual-tasking experiment, they trained the participants with a second-order sequence (e.g., 3 4 2 1 2 3 1 4; numbers 1–4 denoting stimulus positions from left to right) and a tone discrimination task. In one condition, the target stimuli were consistently paired with one particular tone (e.g., the “3” always appeared with a high tone), while in the other condition, the first “3” within the sequence was consistently combined with a high and the second “3” with a low tone. The results revealed implicit sequence learning effects in the first condition, but not in the second condition. The authors concluded that the participants had to learn the associations between the respective SRT-stimulus and the tone before they could exploit the sequential regularity built into the SRT-stimuli.

One last point, important to discuss is the relation between across-task integration on the level of stimuli and responses versus on the level of (sub)task-sets as observed by Kübler et al., (2018, 2021), Strobach et al. (2021) or Hirsch et al. (2021). The focus of these experiments differs from our focus on the level of task-elements. These authors investigate on a more abstract task-set level the effects of changing task-order sets or task-pair sets. For instance, Kübler et al. (2021) or also Strobach et al. (2021) have shown that task-order switches led to additional costs in dual-tasking (see also Luria & Meiran, 2006). This fits with our observation that the participants do not handle the two tasks in dual-tasking as isolated task-sets. Rather, they seem to use a structure of task control that encompasses the (sub)task-sets and their order. While this work suggests across-task integration on the task-set level and our work suggests across-task integration on the element level (stimuli and responses), it is a further issue to test to what extent these two levels operate in separation. For instance, Kübler et al. (2021) assume that task-set order switch costs result from an abstract representation of the task-order sets actively represented in working memory. These representations are thought to contain no information about the specific task components. Rather, the specific task elements are assumed to be maintained separately in working memory. Using task order as an example, Kübler et al. (2022) suggest that bridging across the representations of the two tasks relevant in a dual-task trial may occur at different levels rather independently. They used different tasks with visual and auditory stimuli. In each dual tasking trial both modalities occurred. Participants were faster in trials in which the task order at the level of modalities was the same as in the preceding trial (i.e., again visual task first, auditory second) compared to trials which contained a switch. This effect of task order was observed, irrespective of whether the specific auditory or visual task also repeated from one trial to the next. This might suggest that across-task integration can take place independently on different levels of task representations. Future work on dual-tasking should further address the coupling vs. independence of across-task integration on the level of task representations on the one and the level of task elements (i.e. stimuli and responses) on the other side.

## Concluding remarks

First of all, our findings nicely fit the previous results reported in Pelzer et al., (2021, 2022) and other results from the dual-tasking literature combining a serial reaction time task with a task with random sequence of stimuli and responses (Schmidtke & Heuer, 1997). They add that the generation of a conjoint memory episode seems to be a direct consequence of processing the two tasks within a single trial (Logan, 1988). Our findings suggest that even if participants perform two tasks which are frequently separated by long SOAs, they nevertheless generate conjoint memory episodes. Depending on whether a short or long temporal gap between tasks is more frequent, participants seemed to adapt their processing strategy. The respective processing strategy affected the extent to which they group the processing of the two tasks, thereby modulating the impact of the reactivated memory episode on task performance.

## Data Availability

The merged raw data are available at https://osf.io/42pzc/

## Footnotes

1 The differences between random and regular transitions in the test phase of this experiment were 22 ms (*d* = 0.717) for the fixed and − 19 ms (*d* = − 0.518) for SRTT-tone combinations.

2 In this study, the participants received a visual-manual task together with an auditory-verbal task. Both stimuli were presented simultaneously. The stimuli and responses of both tasks did not follow any regular order.

3 Also in this study, the two stimuli were presented simultaneously.

4 To assess the robustness of the results, we computed Bayes factors with JASP (JASP Team, 2019), using the Bayesian ANOVA framework proposed by Rouder et al. (2017). We conducted a 2 (SOA distr.: SF vs. LF) × 2 (SOA trans.: short-medium vs. long-medium) × 2 (VST-sequence: repetition vs. switch) × 2 (ADT-sequence: repetition vs. switch) Bayesian mixed measurement ANOVA for the RTs in the VST. Here we report the inclusion Bayes factors (BF_inlc_) for the SOA trans. × VST × ADT and the VST × ADT × SOA distr. interaction effect, indicating the change from prior to posterior inclusion odds for the respective effect averaged by all models including this effect. The Bayes factors confirmed the insignificant SOA trans. × VST × ADT interaction effect (BF_inlc_ = 0.085) and the significant effect of the VST × ADT × SOA distrib. interaction (BF_inlc_ = 4.415).

5 Analogous to the VST, to confirm the results in the ADT, we computed Bayes factors with JASP (JASP Team, 2019), using the Bayesian ANOVA framework proposed by Rouder et al., (2017). We conducted a 2 (SOA distr.: SF vs. LF) × 2 (SOA trans.: short-medium vs. long-medium) × 2 (VST-sequence: repetition vs. switch) × 2 (ADT-sequence: repetition vs. switch) Bayesian mixed measurement ANOVA for the RTs in the ADT. Again we report the inclusion Bayes factors (BF_inlc_) for the SOA trans. × VST × ADT and the VST × ADT × SOA distr. interaction effect, indicating the change from prior to posterior inclusion odds for the respective effect averaged by all models including this effect. Again the Bayes factors confirmed both the insignificant SOA trans. × VST × ADT interaction effect (BF_inlc_ = 0.164) as well as the significant effect of the VST × ADT × SOA distrib. interaction (BF_inlc_ = 3.799).
